# Development of a measure to evaluate competence perceptions of natural and social science

**DOI:** 10.1371/journal.pone.0209311

**Published:** 2019-01-02

**Authors:** Caitlin K. Kirby, Patricia Jaimes, Amanda R. Lorenz-Reaves, Julie C. Libarkin

**Affiliations:** 1 Earth and Environmental Sciences Department, Michigan State University, East Lansing, Michigan, United States of America; 2 Department of Entomology, Michigan State University, East Lansing, Michigan, United States of America; Institut Català de Paleoecologia Humana i Evolució Social (IPHES), SPAIN

## Abstract

Interdisciplinary scientific research teams are essential for responding to society’s complex scientific and social issues. Perceptual barriers to collaboration can inhibit the productivity of teams crossing traditional disciplinary boundaries. To explore these perceptual barriers, survey measures related to perceived competence were developed and validated with a population of earth scientists (n = 449) ranging from undergraduates through professionals. Resulting competence scales included three factors that we labeled as Perceived Respect (P_R_), Perceived Methodological Rigor (P_M_), and Perceived Intelligence (P_i_). A Mann-Whitney U test revealed that earth scientists perceived social science/scientists as significantly less competent than natural science/scientists. A multivariate multilevel analysis indicated that women perceived scientists as more intelligent than did men. Working with social scientists and holding an earth science PhD changed earth scientists’ perceptions of social science on multiple scales. Our study indicates that competence in scientific disciplines is a multidimensional construct. Our results from earth scientists also indicate that perceptual barriers towards other scientific disciplines should be studied further as interdisciplinarity in scientific research continues to be encouraged as a solution to many socio-scientific problems.

## Introduction

The complex scientific problems facing today’s world are not easily solved by scholars housed within a single discipline. Environmental and public health problems in particular require an interdisciplinary approach that considers the human context in which problems occur [1, 2, 3]. For example, urban planning requires the incorporation of ecology, sociology, earth sciences, economics, and anthropology to address the biophysical and societal processes that promote the sustainable use of resources [4, 5]. Funding agencies such as the National Science Foundation and National Institutes of Health have increasingly called for interdisciplinarity as a facet of grant-winning proposals in recognition of the need for input from many scientific disciplines in impactful research [6].

Perhaps the largest disciplinary divide among scientists is that between the natural sciences (examining biological, physical, and chemical processes) and the social sciences (examining human dimensions of the world). In discussing the differences between social and natural science, it is important to note that both professions consist of a wide range of disciplines, each with their own methodology, jargon, and culture. Nevertheless, the terms “social science” and “natural science” are used to demarcate funding opportunities, colleges within universities, and categories of research journals. Because the terms natural science and social science are so widely used, they represent meaningful distinctions within the scientific profession.

Natural and social scientists alike have reported benefits from interdisciplinary collaboration with colleagues, including the formation of new perspectives in relation to their own disciplines, the creation of knowledge, and increased relevance of their research in solving environmental problems [6, 7]. However, interdisciplinary research presents many challenges [1, 8]. Methodological and language differences, as well as differences in scales of measurement, are some of the technical issues that interdisciplinary teams face [1, 2, 9, 10]. External factors such as funding and institutional support may be unavailable for interdisciplinary projects since they are perceived to take longer than disciplinary research [1, 11].

Among collaboration participants themselves, perceptual barriers such as a lack of respect also prevent researchers from engaging in projects with colleagues from other disciplines. Perceptual barriers have been well documented over time, especially in the divide between natural sciences and social sciences [1, 8, 9, 12–16]. For example, participants in the 1984 International Geosphere-Biosphere Program, one of the earliest interdisciplinary earth system science programs, noted that both social and natural scientists lacked a mutual respect for each other’s fields [15]. In another study, most social science researchers working in medical research settings felt the need to alter their research practices in order to be viewed as legitimate by their medical research colleagues [17]. In both of these examples, the social sciences were considered necessary for shedding light on human behaviors and their impacts on the environment and personal health, respectively [15,17].

In examining successful interdisciplinary collaborations, Stokols et al. developed a framework of necessary components for interdisciplinary work, including intrapersonal and social factors that foster willingness to engage in interdisciplinary research [18]. However, exactly what personal attributes and perceptions may contribute to the success of interdisciplinary work is not clear [18]. A deeper understanding of perceptual barriers and their prevalence among different scientific disciplines may help promote interdisciplinary collaboration.

### Components of attitudinal barriers

In determining constructs that are important in the formation of attitudes towards a discipline, we draw on research from boundary science, team science, and social psychology. Research in social psychology suggests that warmth and competence are the two primary dimensions across which individuals judge others [19]. Warmth judgements focus on interpersonal characteristics (e.g., trustworthiness, friendliness, empathy, kindness) while competence addresses professional characteristics (e.g., intelligence, power, efficacy, skill) [20]. Here, we focus on competence in considering perceptual barriers towards engaging in collaborations with scientists from other disciplines.

Components of competence within scientific professions include overall respect and other perceptions such as those related to intelligence, objectiveness, methodological rigor, skill, and value to society. Here we discuss these concepts and how they relate to competence in scientific professions. Respect is a multi-dimensional concept encompassing factors such as admiration, listening, sharing, and trust, and is important for the success of any personal or professional relationship [9, 21–23]. Feeling respected as a member of a group is important in forming a sense of group identity and commitment to a group, in this case the scientific profession [24]. Within and outside of the scientific profession, there persists the perception that scientists require high intelligence as measured by IQ in order to succeed [25, 26]. These perceptions may persist despite research that shows many factors beyond intelligence contribute to job success in the sciences [27]. There may also be a perception that social scientists have lower intelligence than natural scientists, supported by discussions on the hierarchy of the sciences, with natural sciences considered more methodologically rigorous than social sciences [28]. Methodological differences are one of the most important distinctions between scientific fields. Many scientists consider methods that are more quantitative and conducted across larger scales to be more rigorous and objective than qualitative and individual-level methods, which are more often used in social sciences [2, 7, 12, 28]. In interdisciplinary projects that span the boundaries between natural sciences and social sciences, social scientists are often required to fit their methodology and results to the theories and expectations of the natural scientists [29]. Natural scientists may be reluctant to acknowledge the presence of subjective value judgements within their research [8], but those who are more critical of their own methodology and recognize subjectivity in their research tend to be more receptive to social science research [12]. These findings emphasize the importance of methodology and objectivity considerations in competence perceptions among scientific disciplines.

The value of science to society can be thought of as an intrinsic sense of worth as demonstrated in monetary or non-monetary ways [30]. A profession has value if it is considered worthwhile to engage in and fund [31]. Value can be added to a profession through external acknowledgments of that profession’s rigor or worth, such as through accreditation processes [32]. The value of the social and natural sciences to society has differed historically, with natural sciences often receiving more federal funding than social sciences [31, 33]. Part of this funding gap could be explained by the need for expensive facilities and equipment in natural science research. For example, all of the $185 million in major research equipment and facilities instruction awards given by the National Science Foundation in 2018 went to natural science fields [34]. However, the Social, Behavioral, and Economic Sciences are also awarded the fewest number of grants of any research area by the National Science Foundation [34].

An international research program examining global environmental change called Future Earth provides an interesting example of value differences. The program included natural and social scientists in order to develop research connected to public policy. Future Earth research proposals for the natural sciences have received more funding than those for the social sciences and publications from the program emphasized goals related to natural science [35]. This funding disparity continued despite the explicit recognition by the program that social sciences could contribute action-oriented research that would allow societal transformations toward sustainability [35]. In this case, natural science research was valued more than social science research when allocating resources and developing publications.

Perceptions of competence may also be influenced by experiential and demographic factors such as gender. For example, when estimating others’ IQs, women give higher estimates and men give lower estimates for the same individuals. Women also give lower self-estimates of IQ than men do [36]. Thus, women may have higher competence perceptions of scientists. Having experience with different disciplines in an educational or workplace setting also impacts individuals’ perceptions. A lack of understanding of science can contribute to a negative attitude towards science on the part of students [37, 38]. Working across disciplinary boundaries with other researchers can promote respect between social and natural scientists for each other’s disciplines, especially when these relationships are durable over multiple years [16, 18]. Interdisciplinary research teams with members experienced in interdisciplinary work are better able to address the differences in their values and methods and proceed towards a common research goal than inexperienced teams [39]. Thus, exposure to and understanding of different scientific disciplines may promote competence perceptions of those disciplines. A similar but opposite effect may be possible, wherein further experience in one’s own field through education or research experience might lessen perceived competence of other disciplines. Competence perceptions should be examined with an understanding of exposure to other disciplines and education in one’s own discipline.

### Current study

The competence perceptions of scientists towards their own discipline or other disciplines has not previously been measured through a validated survey instrument. Documentation of views held by scientists about their own and other disciplines is a necessary first step in understanding barriers to inclusive and interdisciplinary scholarship. The overarching purpose of this study is to develop and validate a measure that can be used to understand perceptions of competence within and between scientific disciplines.

With these challenges in mind, we designed a survey to capture the competence-related perceptions that earth scientists have towards natural and social science, and we validated sub-dimensions of competence measured by this survey. Validating these scales allowed initial exploration of the presence of perceptual barriers towards working across disciplinary boundaries. Since natural scientists have been shown to hold differing views of social and natural science [12–16], we chose members of a natural science discipline (earth scientists) as our sample population for our validation work.

### Research questions

1. Can a valid and reliable measure of competence be developed for use with scientists in the investigation of cross-disciplinary perceptions?
2. What perceptions do earth scientists from a range of career experiences hold about the competence of the social and the natural sciences?
3. What impact do gender, exposure to social science disciplines, and education level have on how earth scientists perceive the social and natural sciences?

To investigate these research questions, we develop and validate scales to measure competence, utilize a Mann-Whitney U test to compare earth scientists’ perceptions of social and natural science, and conduct multilevel multivariate analysis to evaluate the impact of gender and social science exposure on competence perceptions.

## Methods

This human subjects research was approved by Michigan State University IRB #x11-949e.

### Survey development

This study utilized a between-groups experimental design wherein a sample was randomly split. We developed two versions of a survey to measure earth scientists’ competence perceptions of natural and social science. The first survey version solicited competence perceptions of social science/scientists, and the second version solicited competence perceptions of natural science/scientists. The between-groups design was chosen to prevent respondents from modifying their perceptions to reduce any perceived biases about natural or social science.

Each survey version contained a definition of the corresponding type of science for participants to review before beginning the survey. The definition used for social science was adapted from Merriam-Webster [40]: “Social Science can be defined as those disciplines that deal with the scientific study of human aspects of the world.” A similar phrase was used to define natural science [41]: “Natural Science can be defined as those disciplines that deal with the scientific study of the physical and biological aspects of the world.” These definitions were broad enough to encourage respondents to consider overarching disciplines rather than specific fields. Apart from these definitions, the two survey versions contained identical items and phrasing, altered only by specific reference to social or natural science.

Surveys were designed to measure competence, with recognition that competence is a multi-dimensional construct. We conducted a broad search of scales for related constructs, including perceptions of intelligence, value to society, methodological rigor, objectivity, respect, and skill. Measures that we included were gathered from previously validated measures [42], modified from items of related measures [21] to accommodate best practices for writing Likert-type items [43], or developed based on published qualitative studies [12] and descriptions of competence. Where measures were previously validated, which was the case only for a measure of perceived intelligence (P_i_) [42], we maintained the 5-point Likert scale from the original measure. In constructing scales, we elected to use a 4-point scale to force agreement [44]. The use of odd and even scales should not have an impact on our findings [45].

To investigate the impacts of exposure to social sciences on perceptions, participants reported how often and how recently they had worked with social scientists. They also reported whether or not they had completed coursework in the social sciences. In order to ensure consistent understanding across surveys, the social science definition was included on the natural science surveys preceding these demographic questions. Participants were also asked to report their gender, age, natural science degrees held, and ethnicity. See S1 and S2 Surveys for full wording of all survey items.

### Participants

We distributed surveys to individuals attending a professional meeting for earth scientists in 2015. Participants were selected by convenience as they passed by an exhibit hall booth and offered a snack as an incentive to complete the survey. Before participating in the survey, participants were informed that the survey would take about ten minutes and asked to read a document of informed consent as approved by Michigan State University’s IRB #x11-949e. After reading the consent document, individuals’ participation in the survey was considered written consent.

### Statistical analysis

Items from the previously validated intelligence scale P_i_ were recorded on a scale from one to five, with one representing low perceived intelligence and five representing high perceived intelligence. Remaining items were coded from 1–4 indicating strongly disagree (1), disagree (2), agree (3) or strongly agree (4). In the few instances where participants indicated that their level of agreement was in between two points on the scale, the response was coded at the higher level of agreement. One Likert-type question was removed prior to analysis due to multiple possible interpretations of its wording (“Natural/Social science research has few sources of potential bias.”).

Factor analyses were conducted to determine the extent to which items measured unique constructs. The resulting scales failed normality tests, so we utilized a Mann-Whitney U test to examine differences between perceptions related to social sciences and natural sciences. These tests were performed using SPSS version 22 [46].

To test research question three on the impact of gender, exposure to social science, and education level on earth scientists’ competence perceptions, we completed a multilevel multivariate analysis in R using the lme4 package [47]. The mixed-effects model tested for fixed and random effects of gender and social science exposure on natural scientists’ perceptions of social sciences. Gender was input into the model at the first hierarchical level, followed by the social science exposure score. Social science exposure was included as a binary variable that indicated whether respondents had never worked with a social scientist (0) or had worked with a social scientist at some point (1). Involvement in a social science course and working with a social scientist were highly correlated items and could not both be included in the multivariate model. Finally, respondents’ education level was measured as whether they had earned a PhD (1) or not (0) in earth science.

## Results

A total of 518 surveys were collected and are available in the S1 Dataset (social science = 257 and natural science = 261). Respondents who skipped one or more survey questions were excluded from analysis (listwise deletion), with sixty-nine cases of missing data resulting in an analysis of 449 surveys (social science = 216 and natural science = 233). This indicates that complete data were analyzed for 86.7% of all participants, which is above the recommended threshold of 85% for listwise deletion of missing data [48]. Given this result, we felt confident to proceed with our analysis.

Demographics did not differ significantly between the two surveys (Table 1). Respondents were evenly split between male and female, with two individuals reporting their gender as both female and genderqueer. The majority of respondents were white (84.2%). The remaining reported ethnicities for the sample were Asian (6.6%), Latino (6.4%), other (3.6%), Black (3.1%), American Indian (2.1%), and Native Hawaiian (0.4%). Based on these demographics, we conclude this sample is very similar to the larger population of earth scientists [49]. The age range of our sample was 18–83, with an average of 31.5±13.9 years. Roughly even percentages of individuals were undergraduate students (35.6%), graduate students (35.4%), and non-student professionals (29%). Undergraduate students are included as scientists in our sample because students in earth and environmental sciences are likely to engage in research as undergraduates [50], and thus are actively participating in the scientific process. The fact that these students were attending a professional conference demonstrates engagement in the earth science profession. In addition, Bachelor’s degrees are sufficient for obtaining employment in the environmental science field [51]. Nearly half of the sample held a Bachelor’s degree in natural science, with about 1 in 5 individuals holding a PhD. Of individuals who reported their education levels, a majority were pursuing or held a degree in earth science, with 2.4% of respondents holding or pursuing a natural science degree in another concentration such as biology or chemistry. Finally, 78.0% and 61.3% of respondents indicated prior work with social scientists and prior coursework in social science, respectively.

**Table 1:** Demographics of survey respondents. Respondents (n = 449) were earth scientists attending a 2015 professional conference. Approximately half of the respondents (n = 233) reported their perceptions of natural science and the other half (n = 216) reported their perceptions of social science.

Demographics	All Respondents	Natural Science Survey	Social Science Survey
Female	50.7%	52.2%	50.7%
White	84.2%	82.2%	86.2%
Age (years)	31.7 ± 13.7	32.0 ± 13.4	31.5 ± 13.9
SS coursework^1^	65.0%	68.8%	61.3%
SS exposure^2^	82.2%	86.3%	78.0%
Pursuing undergraduate degree^3^	35.6%	34.6%	36.6%
Pursuing graduate (Master’s or PhD) degree^3^	35.4%	34.6%	36.1%
Hold B.S.^3^	39.3%	40.8%	37.9%
Hold Master’s^3^	20.4%	20.6%	20.1%
Hold PhD^3^	19.4%	21.4%	17.4%

^1^Participants who have taken a social science course

^2^Participants who reported having worked with a social scientist

^3^Degrees held or pursuing were in the natural sciences; all degrees held or pursuing are reported, not only the highest degree.

### Scale validation

A simple confirmatory factor analysis with maximum likelihood extraction was used to validate the Perceived Intelligence (P_i_) scale for our sample. As expected from Bartnek et al. [42], the five intelligence items correlated with each other with Pearson coefficients >0.3, suggesting that a factor pattern was present in the data [52]. Anti-image correlation diagonals were all >0.5, which also indicates that factor analysis is appropriate to use on the items [52]. The Kaiser-Meyer-Olkin measure was 0.858, well above the recommended value of 0.6 [52]. Based on eigenvalues >1.0 and scree plot analysis, a single factor emerged for the intelligence scale that explained 58.7% of the variance (Table 2) [53]. Cronbach’s alpha, a commonly used measure of internal consistency, for the P_i_ scale was 0.874. Values above 0.8 are widely considered adequate, with values above 0.5 appropriate for psychological constructs and initial development of scales [52].

**Table 2:** Factor loadings for intelligence survey items. Results are for a simple confirmatory factor analysis with no rotation. Factor loadings >0.32 indicate each item loads onto the intelligence factor [54] Intelligence items were taken from Bartneck et al. [42].

Survey Item	Factor Loadings
Incompetent-Competent	0.592
Ignorant-Knowledgeable	0.744
Irresponsible-Responsible	0.832
Unintelligent-Intelligent	0.816
Foolish-Sensible	0.820

An exploratory factor analysis with maximum likelihood extraction and varimax rotation was conducted on all other survey items. A bivariate correlation indicated a factor pattern was present, as each item correlated significantly with at least one other item at >0.3 [52]. All survey items exhibited diagonals >0.5 in the anti-image correlation matrix, also indicating that all of our questions should be included in the analysis. The Kaiser-Meyer-Olkin measure of sampling adequacy was above the 0.6 minimum value at 0.941 [52].

Factor analysis for our developed survey items resulted in the emergence of two separate factors interpreted as Perceived Respect (P_R_) and Perceived Methodological Rigor (P_M_) based on eigenvalues >1.0 and scree plot analysis [53]. The first factor explained 38.8% of the variance and the second factor explained 11.0% of the variance for a total of 49.8% (Table 3). The P_R_ scale had a Cronbach’s alpha of 0.932. The Cronbach’s alpha of the P_M_ scale was 0.646.

**Table 3:** Factor loadings and sources for survey items. Exploratory factor analysis with varimax rotation resulted in a Perceived Respect scale, P_R_, and a Perceived Methodological Rigor scale, P_M_. Factor loadings <0.32 are suppressed [54].

Survey Item	P_R_ Factor Loadings	P_M_ Factor Loadings	Source
Social/natural scientists have admirable talents and skills	0.562		[21]
Social/natural science research is beneficial for society	0.642		This study
I respect the work of social/natural scientists	0.730		This study
Social/natural scientists are worth listening to	0.786		[21]
I admire the work that social/natural scientists do	0.836		[21]
Social/natural science research is worthwhile	0.863		This study
I value the knowledge gained from social/natural science research	0.867		This study
More funding should be allocated for social/natural science research	0.763		This study
Reliable conclusions can be drawn from social/natural science studies	0.642		[12]
Social/natural scientists are members of a respect worthy group	0.782		[21]
The implications of social/natural science research are often unclear		0.641	This study
Many social/natural science studies are difficult to reproduce		0.686	[12]
Social/natural science research contains many sources of potential error		0.327	This study
Many assumptions are required to perform social/natural science research		0.419	[12]
It is difficult for social/natural scientists to be objective		0.418	[12]

### Earth scientists’ perceptions of social and natural science

Scores for each of the three measures (P_i_, P_M_, P_R_) were computed by summing the scores from each individual. Negatively worded items were reversed such that higher scores on each scale indicated a more favorable perception of P_i_, P_M_, or P_R_. Perceived Respect P_R_ and Perceived Methodological Rigor P_M_ were each created on a 4-point Likert scale, with Perceived Intelligence P_i_ retaining the 5-point Likert scale with which it was created and validated [42]. When reversing items, we scaled them from 0 to 3. Each scale also had a different number of items. Thus, each scale had a different range of possible scores. P_M_ scores ranged from 0–15. Possible P_R_ scores ranged from 10–40 and P_i_ scores ranged from 5–25.

Mean scores across NS and SS participants were calculated for each measure. Across both the social science (SS) and natural science (NS) surveys, P_R_ scores had the highest relative value (overall 87% of maximum; Table 4). P_i_ scores were also relatively high overall (overall 84% of maximum). This indicates that our sample respects science/scientists, regardless of discipline, and believes scientists to be intelligent. The P_M_ scale scores were the lowest relative to their scale range (overall 45% of maximum), indicating that our sample feels that scientific methodology in general is not perfectly objective or without flaws.

**Table 4:** Means of factor scores displaying standard deviation and Mann-Whitney U test showing the difference between SS and NS scale scores. Higher values indicate more favorable perceptions of respect (P_R_), methodological rigor (P_M_), or intelligence (P_i_).

Scale	Scale Range	Overall Value	SS Value	NS Value	Mann-Whitney U	Z	p-value
P_R_	10–40	35.0±4.7	32.4±4.7	37.5±3.1	10441	-11.76	<0.001
P_M_	0–15	6.7±2.3	6.0±2.4	7.3±2.1	20355	-6.45	<0.001
P_i_	5–25	21.0±3.4	20.2±3.4	21.8±3.1	22802	-5.65	<0.001

Scores failed tests of normality, so a non-parametric test was chosen to analyze differences between SS and NS scale scores. We utilized a Mann-Whitney U test to compare participants’ scores for all three scales across the groups of NS and SS (Table 4). To avoid type 1 error, a critical value of 0.017 was used to achieve the 0.05 significance level according to the Bonferroni correction; each scale surpassed this significance level. For all three scales, social science/scientists were perceived as less competent than natural science/scientists (Table 4). The difference in the average P_R_ score was 5.1 points out of 30, representing the largest disparity between perceptions of SS and NS.

In examining our third research question about the impact of gender, social science exposure, and education level on competence perceptions, we examined the scale scores across these groups on the SS survey (Table 5). Individuals who reported having worked with a social scientist had the highest average scores on the Perceived Respect scale P_R_ and Perceived Methodological Rigor scale P_M_. Female respondents reported the highest average scale scores for Perceived Intelligence P_i_. The lowest average scores on all three scales were from respondents with PhDs in earth science.

**Table 5:** Social science scores across gender, social science exposure, and education level. Higher values indicate more favorable perceptions of respect (P_R_), methodological rigor (P_M_), or intelligence (P_i_). Standard deviation is given with the average scale scores. Items with superscripts of the same letter are significantly different from one another (p<0.05).

Independent Variable		P_R_ Scale Score	P_M_ Scale Score	P_i_ Scale Score
Gender	Female	32.7 ± 4.8	6.1 ± 2.5	20.8 ± 2.7^a^
	Male	32.0 ± 4.7	5.8 ± 2.2	19.5 ± 3.6^a^
SS Exposure	Have not worked w/SS	31.4 ± 4.4^b^	5.8 ± 2.0^c^	19.6 ± 3.5^d^
	Have worked w/SS	33.1 ± 4.9^b^	6.2 ± 2.5^c^	20.7 ± 3.3^d^
Education Level	Do not hold PhD	32.4 ± 4.6	6.0 ± 2.4^e^	20.5 ± 3.2^f^
	Hold PhD	30.9 ± 5.3	5.5 ± 2.2^e^	18.8 ± 4.3^f^

In the multilevel multivariate analysis, gender, social science exposure, and education level had a significant impact on at least one of the scale scores (Table 5). Male earth scientists rated social scientists as less intelligent (β = -1.081, SE = 0.473, p<0.05) than did female earth scientists by an estimated 1.08 points on the 20-point P_i_ scale. However, the scales of P_R_ (β = -0.521, SE = 0.490, p = 0.29) and P_M_ (β = -0.332, SE = 0.480, p = 0.49) were not impacted by individuals’ genders. Respondents who had worked with social scientists rated them as more intelligent (β = 1.096, SE = 0.479, p <0.05) by an estimate of 1.10 on the 20-point P_i_ scale. Respondents who worked with social scientists also had higher P_R_ scores (β = 2.094, SE = 0.497, p < .001) by an estimate of 2.09 on the 30-point P_R_ scale. Respondents with earth science PhDs perceived social scientists as less intelligent (β = -1.879, SE = 0.625, p < .01) and less methodologically rigorous (β = -1.854, SE = 0.650, p < .01) than respondents without PhDs. Model goodness of fit was increased with the inclusion of gender, social science exposure, and education level (χ^2^(6) = 23.51, p<0.001).

A multilevel multivariate analysis was carried out for the results of the natural science survey as well, with social science exposure resulting in no significant difference for any of the scales. However, female earth scientists again perceived natural scientists as more intelligent (β = -0.906, SE = 0.359, p < .05) than did male scientists and also reported higher P_R_ scale scores (β = -0.753, SE = 0.363, p < .05). Respondents who held a PhD in earth sciences perceived natural scientists as more intelligent (β = 0.988, SE = 0.432, p < .05) than did non-PhD holders. While statistically significant, the average differences for competence perceptions of natural science were smaller than those for social science. A goodness of fit test for the model indicated that adding gender, social science exposure, and PhD degree did not significantly improve model fit (χ^2^(9) = 15.742, p = 0.08).

## Discussion

Among increasing calls for interdisciplinary work to solve today’s most complex scientific issues [55, 56], this study seeks to create a measure for understanding scientists’ perceptions of natural and social science/scientists. Investigation of Perceived Respect (P_R_), Perceived Intelligence (P_i_), and Perceived Methodological Rigor (P_M_) as components of competence indicates that earth scientists perceive social science/scientists as less competent than natural science/scientists. Scales generated in this study can be used to measure the extent and pervasiveness of potential perceptual barriers to interdisciplinary collaboration across different scientific disciplines. This work also improves our understanding of different dimensions of perceived competence in the workplace by creating and validating scales of competence constructs. Because of the importance of competence in professional and collaborative group settings [20, 22], there is value in understanding these perceptions within scientific disciplines towards other disciplines.

Earth scientists’ competence perceptions of natural science and social science were significantly different, suggesting important implications for collaboration potential. Earth scientists’ scores on the three scales of Perceived Respect (P_R_), Perceived Methodological Rigor (P_M_), and Perceived Intelligence (P_i_) of social science and social scientists were significantly lower than those for natural science and natural scientists. These survey results provide a quantitative confirmation of the challenges of affording the same value to methods and findings in the social sciences as are often found in the natural sciences [8, 12, 31, 33, 35]. The competence scales also provide a more detailed understanding of the social and attitudinal barriers described in the formation of interdisciplinary teams [15, 17, 18].

In our study, female respondents viewed both social and natural scientists as significantly more intelligent than did male respondents. Females are more likely than males to trust the expertise of scientists [57], with our results indicating that this may include those from disciplines other than their own. While not studied extensively, there is evidence outside of our study to suggest that females are more drawn to interdisciplinary fields and projects than are males [58]. Given these prior results and our current study, the impact of gender on interdisciplinary work merits additional exploration.

Earth scientists with experience working with social scientists perceived social science/scientists as more competent than did respondents lacking such experience. However, these scale scores were still lower than those for natural science/scientists, suggesting that there may still be some perceptual barriers to working across disciplines. Developing respect for others’ disciplines may require ongoing involvement in a multidisciplinary project over several years [16], and even then individuals tend to exhibit more positive attitudes towards subject matter that they are more familiar with. This is known as the mere exposure effect and is a consistent and well-studied phenomenon where increased familiarity with a certain stimulus improves one’s attitude towards that stimulus [59, 60]. The mere exposure effect has been shown to have an impact on attitudes towards scientific constructs such as journal rankings [61].

The mere exposure effect contextualizes earth scientists’ competence perceptions of social science compared to natural science. Natural science is more familiar to earth scientists than social science and that familiarity alone may alter earth scientists’ competence perceptions of each field. More familiarity in natural science, as indicated in this study by having attained a PhD, is also linked to perceiving social science as less competent. The mere exposure effect also predicts the improvement of competence perceptions via working with social scientists, and suggests that one way to alter perceptions of social science by natural scientists is to foster interdisciplinary collaborations. This can and is being achieved via funding opportunities for interdisciplinary projects [55]. Engagement in social science courses is another avenue through which natural scientists may gain exposure to the social sciences. Although not included in the multivariate model because of its correlation with working with a social scientist, having taken a social science course was highly correlated with the P_R_ scale on the SS survey. Universities should promote interdisciplinary coursework to produce scientists who are willing and prepared to work across traditional disciplinary lines [39, 62].

Further work in understanding relations between scientific professions might also consider perceptions of warmth, a measure of friendliness, trustworthiness, empathy, and kindness [20]. Capturing measures of warmth in relation to disciplines may build on our scales that explore constructs related to competence. Warmth contributes to building social capital and maintaining professional relationships [20] and impacts affective and behavioral reactions important for group work [23]. Warmth is also important in interpersonal relationships, such as a working professional relationship. Expanding future work to include measures of warmth may offer a deeper understanding of interdisciplinary collaborations and point to additional potential avenues for improving such relationships.

In a scientific world with increasing calls for interdisciplinary work, competence perceptions have important potential impacts on effective collaboration across disciplines. A precursor to successful collaboration is a basic level of respect for the individual performing that work and the research that they will perform. In fostering interdisciplinary collaborations, institutions and funding agencies should be aware of perceptual barriers related to competence and its sub-dimensions of intelligence, methodological rigor, and respect. Interdisciplinary team leaders could also utilize team members’ competence perceptions in planning team-building exercises, which are important to the success of diverse research teams [63, 64]. Competence perceptions might also be considered in integrating social science coursework into natural science degree requirements. Examining these perceptions provides opportunities to improve willingness to work across disciplinary boundaries, allowing researchers to find solutions for society’s most complex, interdisciplinary problems.

## Limitations and future work

Our study population consisted primarily of early-career earth scientists, and earth scientists represent only one small discipline of the natural sciences. Future studies that expand the experience level and discipline of respondents will be better able to make conclusions about competence perceptions of other disciplines. It was also outside the scope of this study to survey social scientists’ perceptions of natural scientists. In the creation of our scales, the items that scaled onto the P_M_ (Perceived Methodological Rigor) scale were all reverse coded due to their wording suggesting low methodological rigor. Future studies that utilize, expand upon, or otherwise improve this scale might consider mixing positively and negatively worded items within both the P_M_ and the P_R_ (Perceived Respect) scales.

This paper is not concluding that natural scientists such as earth scientists are responsible for the majority of perceptual barriers between natural and social sciences, nor that natural scientists are inherently more likely to have less favorable perceptions of other disciplines. Social scientists might also have more favorable attitudes towards social science than natural science because of their familiarity with their own field. Within either silo of science, scientists from different sub-disciplines, such as chemistry and ecology or history and sociology, may also exhibit these relatively less favorable attitudes towards other disciplines. Future work should consider competence perceptions across more disciplines to more fully understand how these perceptual barriers might impact interdisciplinary projects of all types. Because this study was designed to create and validate scales, it was outside of the scope to survey across many groups and is both a limitation of the present study and an opportunity for future work.

## Supporting information

S1 Survey Social science competence survey. The full social science version of the survey and given to participants.
(DOCX)

S2 Survey Natural science competence survey. The full natural science version of the survey and given to participants.
(DOCX)

S1 Dataset Natural and social science dataset. Coded dataset with responses from both the natural and social science surveys.
(XLSX)
