# Supplementary material for: Development of a measure to evaluate competence perceptions of natural and social science
Source: PLoS One. 2019 Jan 2;14(1):e0209311. doi: 10.1371/journal.pone.0209311 (PMC6314610; doi:10.1371/journal.pone.0209311)
Supplement: S2 Survey — The full natural science version of the survey and given to participants. (DOCX) [file pone.0209311.s002.docx]

**S2. Survey**

***Please use the following definition of natural science to answer the questions below as honestly as possible.***

**NATURAL SCIENCE can be defined as those disciplines that deal with the scientific study of physical and biological aspects of the world.**

**1. Rate your agreement with each of the following statements.**

|  | Strongly Agree | Agree | Disagree | Strongly Disagree |
| --- | --- | --- | --- | --- |
| Natural science research contains many sources of potential error. | A | B | C | D |
| Natural scientists have admirable talents and skills. | A | B | C | D |
| Many assumptions are required to perform natural science research. | A | B | C | D |
| Natural science research is beneficial for society. | A | B | C | D |
| Natural science research has few sources of potential bias. | A | B | C | D |
| I respect the work of natural scientists. | A | B | C | D |
| It is difficult for natural scientists to be objective. | A | B | C | D |
| Natural scientists are worth listening to. | A | B | C | D |
| I admire the work that natural scientists do. | A | B | C | D |
| Natural science research is worthwhile. | A | B | C | D |
| I value the knowledge gained from natural science research. | A | B | C | D |
| More funding should be allocated for natural science research. | A | B | C | D |
| Reliable conclusions can be drawn from natural science studies. | A | B | C | D |
| Natural scientists are members of a respect worthy group. | A | B | C | D |
| The implications of natural science research are often unclear. | A | B | C | D |
| Many natural science studies are difficult to reproduce. | A | B | C | D |

**2. Please rate your impression of NATURAL SCIENTISTS according to the scales below.**

| Incompetent | 1 | 2 | 3 | 4 | 5 | Competent |
| --- | --- | --- | --- | --- | --- | --- |
| Ignorant | 1 | 2 | 3 | 4 | 5 | Knowledgeable |
| Irresponsible | 1 | 2 | 3 | 4 | 5 | Responsible |
| Unintelligent | 1 | 2 | 3 | 4 | 5 | Intelligent |
| Foolish | 1 | 2 | 3 | 4 | 5 | Sensible |

**The following questions ask about the SOCIAL SCIENCES.**

**SOCIAL SCIENCE can be defined as those disciplines that deal with the scientific study of human aspects of the world.**

**3. How often do you work with social scientists in a professional capacity?**🞎 Frequently 🞎 Sometimes 🞎 Never

**4. When was the last time you worked with a social scientist in a professional capacity?**

| 🞎 Within the last 6 months 🞎 Within the last year 🞎 Within the last 5 years |
| --- |
| 🞎 More than 5 years ago 🞎 Never  **5. Have you ever taken a course or courses in the social sciences?**  🞎 No 🞎 Yes *If Yes,* *please elaborate:*__________________________________________ |
|  |
|  |

***Please respond to the demographic questions below. Your responses will be kept strictly confidential.***

| 1. How many undergraduate courses in *Natural Science* have you completed? ____ |
| --- |
| 1. How many graduate courses in *Natural Science* have you completed? ____ |
| 1. Are you currently pursuing any of the following in *Natural Science*?   🞎BA/BS 🞎MA/MS 🞎PhD |
| 1. Do you hold any of the following in *Natural Science*? 🞎BA/BS 🞎MA/MS 🞎PhD |
| 1. When did you receive your highest degree in *Natural Science*? __________ |
| 1. How many years have you worked as a *professional Natural Scientist?* DO NOT include years in which you were engaged primarily in undergraduate and graduate education. If you have no professional experience, write NONE: _____________ |
| 1. What is your current job title? ______________________________________________________ |
| 1. What is your primary field of study? ______________________________________________________ |

**I. What is your AGE?** _________

**J. Is English your native language?** 🞎 Yes 🞎 No

*If no, indicate your native language*:__________________

**K. Which of these terms best describes your GENDER IDENTITY? CHOOSE ALL THAT APPLY.**

🞎 Male 🞎 Female 🞎 Transgender male to female 🞎 Transgender female to male 🞎 Agender 🞎 Genderqueer 🞎 Something else. Please specify: _______________

| **L. What is your ETHNICITY:**  **CHOOSE ALL THAT APPLY.** | **M. Do you self-identify as having any of the following DISABILITIES?** **CHOOSE ALL THAT APPLY.** |
| --- | --- |
| 🞎 American Indian/Native American | 🞎 I do not identify as having any disability |
| 🞎 Asian/Asian American | 🞎 Blind/visual disability |
| 🞎 Black/African/African American | 🞎 Deaf/hard of hearing/hearing disability |
| 🞎 Latino(a)/Hispanic | 🞎 Physical/orthopedic disability |
| 🞎 Native Hawaiian/Pacific Islander | 🞎 Learning/cognitive disability |
| 🞎 White/Caucasian | 🞎 Vocal/speech disability |
| 🞎 Other ________________ | 🞎 Other ________________ |

|  |
| --- |

**N. Would you like to tell us anything else about yourself?**
